# Obstetrics, and Diseases of Women and Children

**Published:** 1859-11

**Authors:** Emil Fischer


### OBSTETRICS, AND DISEASES OF WOMEN AND CHILDREN.

BY EMIL FISCHER, M.D.

I.—*On Amputation of the Shoulder-blade as a Method of Embryotomy.* By Prof. Levy.

The author remarks that it would be a very desirable progress in the obstetric art if embryotomy could be wholly dispensed with, but that at the present time a total abolition of its practice was impossible. Among the cases of labor which may render this operation necessary, there are some, of not rare occurrence, in which, on presentation of the shoulder with descent of the arm, turning, at the right time, has been neglected, and in consequence of it the shoulder, with the side of the chest, is pressed down so firmly into the pelvis, while the uterus, in a state of abnormal contraction, incloses the child so tightly that turning is quite impracticable. In such cases perforation and evisceration of the thorax has been commonly recommended; some, for instance Dubois, advise decapitation of the child; others, among them Cederschjoeld, breaking or cutting through of the spine; and quite recently, Heyerdahl and Faye recommend sawing it through by means of a chain-saw. The proposition of the author is made with the object of rendering turning, with the least possible mutilation of the child, practicable, and is only an improvement upon the inadequate attempts at operation which have been made since old times. While already in the remotest ages obstetricians endeavored to remove the prolapsed arm partly by twisting it off, partly by different modes of amputation, without gaining real advantage, it is possible to obtain, at least in many cases, a favorable result by severing the scapula from the body together with the arm. It is sufficient to contemplate the size of the arch which is formed at the skeleton of the child by the spine of the scapula, the acromion, and the clavicle, in order to become aware of the advantage gained in this way; but it becomes still more evident, if the arm with the respective shoulder-blade and clavicle is removed in the dead body, and the two halves of the thorax are compared with each other. In well-developed bodies of children, the author found that the breadth of the shoulders was diminished as much as one inch and a half by this operation. The breadth of the shoulders being thus reduced, and additional room being gained by the removal of the whole length of the scapula, it will not be impossible to introduce the hand along the chest, and to perform turning without any fear of the danger which arises from the sharp margins and points of bones after embryotomy.

The operation is executed with but little difficulty. The operator draws the prolapsed arm tightly downward, and thereby removes the anterior or posterior margin of the scapula so much from the thorax that it forms a distinct projection, and can be easily felt through the soft parts. This being done, an assistant fixes the arm in this extended position, while the operator, having applied some fingers of his left hand to the projecting margin of the scapula, introduces with his right hand Smellie's scissors along them, and divides the soft parts within and along the margin of the scapula. Guarded by the shoulder-blade itself, the instrument easily penetrates, by small sections, between the subscapular and great serrate muscles, and if the arm is now gradually turned in proper directions, the partly amputated shoulder-blade is removed so much from the body that the section of the soft parts at its opposite margin can be, under protection of the fingers, easily accomplished. At last the clavicle, which is already partly severed from the thorax by the tractions made at the hand, is removed by a single cut of the scissors.

Within the last few years the author has performed this operation, with subsequent turning upon the feet, in two cases. In a third case, complicated with narrowness of the pelvis, the chest was compressed to such an extent that the ribs, being forcibly bent, hindered the introduction of the hand; the author overcame the impediment by subcutaneous section of some of the ribs, the great flaccidity of the integuments of the fœtus inducing him to adopt this method, by which he was able to avoid the danger of injury by the sharp margins of the bones which would have otherwise projected. Dr. Raw, also, executed the same operation successfully in a case of embryotomy; and a Norwegian physician informed the author of a case in which, believing that he was cutting through the neck, he, by mistake, severed the scapula from the body, and was in consequence able to perform easily the operation of turning.—(*Bibliothek für Laeger*, Bd. x., p. 431; *Schmidt's Jahrbücher*, April, 1859.)

II.—*Contribution to the History of Extra-Uterine Pregnancies.* By PROFESSOR HECKER.

The researches of Prof. Hecker are founded upon a great number of cases, collected from various authors. They were undertaken with the object of investigating the different varieties of extra-uterine pregnancy separately, which in the statistics, published up to the present time, have been generally mixed together. The author occupied himself principally with the study of tubal, interstitial, and abdominal pregnancy.

*Tubal pregnancy.*—According to the opinion generally accepted, this variety is the most frequent. Prof. Hecker thinks, on the contrary, that it is rarer than abdominal pregnancy; the latter amounts to 132 in the tables which he has compiled by collecting indifferently all the cases of extra-uterine pregnancy on record, while tubal pregnancy is represented by only 64 cases. Prof. Hecker, moreover, does not share the widely-spread opinion, according to which tubal pregnancy is more frequent in multiparæ than in primiparæ; of 53 cases of tubal pregnancy there are 16 which relate to primiparæ, and 37 which refer to multiparæ. The proportion of multiparæ to primiparæ is here even smaller than for pregnancies in general.

The tubal pregnancy is preceded, in a great number of cases, by a long interval, during which the woman had not conceived, and this remark applies as much to primiparæ as to multiparæ. These two anomalies seem to be the effect of one and the same cause, which will be mentioned by-and-by.

In all the cases collected by Prof. Hecker, with one exception, the tubal pregnancy terminated fatally. The only case of cure on record has been reported by Prof. Virchow, and relates to a woman who presented in June and July, 1848, all the symptoms of an internal rupture; she recovered, and died afterwards, on the 17th of July, 1852, of gangrenous pneumonia. The fœtus, in this case, had not left the tube, although the latter had been ruptured. This termination has, perhaps, presented itself in other cases, but the diagnosis remained uncertain in them. It is also possible that, under some circumstances, the fœtus may die at an early hour, and undergo the cretaceous transformation. The cases where the tubal pregnancy transforms itself into a secondary abdominal, or a sub-peritoneo-pelvic pregnancy, are not any more favorable than those where this transformation does not take place.

The rupture of the tube has been fatal, in two-thirds of the cases, at the end of from six to twenty-four hours; only in one case ten days passed before death occurred. The rupture was produced 26 times in the first two months, 11 times in the third, 7 times in the fourth, and once in the fifth month.

The fœtus occupied the left tube 37 times, and only 27 times the right tube. These numbers confirm an opinion, adopted long ago.

The tubal pregnancy is often accompanied by various adhesions of the internal genital organs, produced by false peritoneal membranes. These adhesions occupy, according to Prof. Virchow, mostly the left side, and it seems rational to attribute to them an important part in the etiology of tubal pregnancy; to the same adhesions also the sterility seems to be owing which often precedes tubal pregnancy. The influence of these bands was especially manifested in a case of Prof. Virchow, in which the fœtus had developed itself at a distance of one inch and a half from the abdominal orifice of the right tube, between two points glued to the neighboring parts by peritoneal adhesions.

In speaking of the pathological anatomy of tubal pregnancy, Prof. Hecker relates a very curious case of Dr. Drejer. The fœtus had developed itself in the right tube, which was inserted at the point of junction of the body and neck of the uterus; the corpus luteum, measuring half an inch in diameter, occupied, on the contrary, the left ovary. It must, therefore, be assumed that the ovum, after having enterted the uterus through the left tube, had slipped afterwards into the orifice of the right tube, which occupied a much lower place than usual. It is, in fact, impossible to conceive that the right tube could have reached as far as the left ovary, and embraced it at the moment when the ovum fell. Prof. Scanzoni has, besides, published a case of pregnancy in a rudimentary cornu uteri which can be explained only by an analogous migration of the ovum.

In 40 cases of tubal pregnancy, the uterus was augmented in size in 24; the decidua existed 25 times; three times the uterus had developed itself to a certain degree without there being any decidua discoverable. In two cases only it is said that the uterus was not at all modified.

2. *Interstitial pregnancy.*—Dr. Hecker could collect but 26 cases of it. The irregularities in fecundation, already pointed out in relation to tubal pregnancy, are quite as remarkable with reference to this variety. The termination has constantly been fatal, and death has always ensued very shortly after the rupture, (in six to twenty-six hours in the majority of the cases.) The rupture took place once at the end of four weeks, twice at the end of two months, 12 times in the third month, 3 times in the fourth, and once in the fifth; 6 times, finally, it occurred only in the eighth or in the ninth month. Also here the pregnancy occupied oftener the left side (17 times in 24 cases) than the right, and the internal genital organs were often attached to the surrounding parts by peritoneal bands. The development of the uterus was generally much more considerable than in tubal pregnancy. The cavity occupied by the ovum was always separated from the cavity of the uterus by a complete partition, and the tube itself was mostly impermeable.

3. *Abdominal pregnancy.*—It is, like tubal pregnancy, most frequently observed in women between the age of thirty and forty years. Multiparæ are more liable to it than primiparæ; the proportion is 90 to 15 in the cases collected by Prof. Hecker, (the usual proportion of multiparæ to primiparæ being 3½ to 1.) The irregularities in the conceptions are again distinctly pronounced in a great number of the women.

The mortality of abdominal pregnancy is not as considerable as is generally believed; among 132 cases Prof. Hecker found 76 cures and 56 deaths, which gives a mortality of 42 per cent.

In the great majority of the cases, the pregnancy arrived at least at its habitual term, and the child could, in exceptional cases, live much beyond this period. In a case of Grossi, the movements of the fœtus were perceived by the mother during twenty-three months; moreover, in some fœtuses, mummified under these conditions, perfectly developed teeth have been found in the jaws. There is even a case reported by Dr. Schmitt, in which, as it is stated, the pregnancy lasted three years, and gastrotomy having been performed after the death of the mother, a living child was removed. This fact seems nearly incredible, and still the circumstances under which it has been observed hardly permit us questioning its authenticity.

The pains which the patients feel so often at the end of an abdominal pregnancy seem to be owing, at least in a certain number of cases, to contractions of the abnormal sac formed round the ovum, and not to contractions of the womb. In a certain number of cases, in fact, the presence of unstripped muscular fibres in the wall of this sac has been proved; the existence of these contractions has been, moreover, demonstrated by Prof. Hohl in an unequivocal manner. In his case the sac, which was seated behind the posterior cul-de-sac of the vagina, and near the posterior wall of the pelvis, could be easily felt, and was observed to contract distinctly, and in a very energetic manner, during the pains. On autopsy the coats of the ovum were found to contain many organic muscular fibres.

Among the 76 cases of cure, there were 28 where the ovum was evacuated by the rectum, 17 where it was mummified, 15 where it was discharged through the anterior wall of the abdomen, 11 where gastrotomy was performed, and 3 where the child was extracted through the vagina. In the remaining two cases no precise information could be obtained.

The 56 fatal terminations distributed themselves in the following manner:—death by exhaustion, 18 times; by peritonitis, 12 times; by rupture and hemorrhage, 7 times; by internal strangulation, twice; by dropsy, once; in consequence of operations, 12 times; undetermined cases, 4.

In 26 operations, there would have been 14 cures and 12 deaths; but it is more than probable that these numbers are too favorable. Gastrotomy would count 11 successes and 5 deaths; in the 11 cures, the fœtus was dead 8 times before the operation, 3 times it was alive at the moment of the operation, and twice it was extracted alive. In the 5 cases which became fatal to the mother, the fœtus was dead in 2; 3 times it was extracted alive.

The extraction by the vagina counts 3 cures and 2 deaths; puncture alone, or associated with cauterization, has been fatal in 4 cases; in one case, finally, the fœtus was extracted from the bladder by the hypogastric section, and the mother died.

These numbers lead to the conclusion that, in general, all operative interference, in cases of abdominal pregnancy, ought to be renounced; there is hardly any indication for it except when it becomes a question whether to save a living child, arrived at term.—(*Monatsschrift für Geburtskunde*, February, 1859.)

III.—*On the Induction of Premature Labor by Uterine Catheterism.* By PROF. BRAUN, of Vienna.

Professor Braun, of Vienna, states that he has for several years given a preference to this mode of inducing premature labor; inasmuch as it is very certain, operates rapidly and safely, brings on the pains with gradual energy, gives rise to no ill consequence, such as congestion or injury of the uterus, or detachment of the placenta, and is performed by the single application of a simple instrument. One disadvantage of the procedure is, that the membranes may become somewhat easily ruptured, especially in primiparæ. In order to prevent this accidental rupture, the author softens in hot water the end of a well-oiled catgut bougie, a foot long, and from two to three lines thick, and passes it along the index finger with a twisting movement into the uterine cavity, until only a portion, equal to two fingers' breadths, remains in the vagina. The bougie so passed always excites pains in from six to twenty hours, does no injury to the membranes, and is to be removed only just before the discharge of the waters, or the birth of the child. The employment of a gum catheter, having a very thin, flexible stilette, is usually also attended with good effect. Its application is difficult, however, when the vagina is narrow, and deviates from the pelvic axis. During the session 1857 and 1858, Professor Braun employed catheterism twelve times, nine children being born alive, and six dead—three being twin-births. Of the mothers eight recovered, and four died during the puerperal state, pneumonia, tubercle, and Bright's disease having been respectively the causes of death. The labor was terminated at an average period of twelve hours after the introduction of the catheter. Brief accounts of these twelve cases are subjoined.—(*Wien medizin. Wochenschrift*, 1858, No. 46; *Med. Times and Gazette.*)

IV.—*On the Treatment of Uterine Hemorrhage by Ruta and Sabina.* By PROF. BEAU.

Professor Beau attributes to ruta as much of a special relation to the uterus as there exists between digitalis and the heart, nux vomica and the cerebro-spinal system, cantharides and the bladder, and between belladonna and the muscular system. In this specific action upon the womb ruta is allied to sabina and ergot; the latter, however, Professor Beau does not consider as reliable in its effect as the two former medicines, particularly if they are given combined with each other; in cases, therefore, where the indication is perfectly plain, and urges to active interference, he does not lose time by experimenting with the unreliable ergot, but prescribes at once the following combination:—

℞.—Pulv. Rutæ, gr. iij; Pulv. Sabinæ, gr. j; Syr. Spilis, q. s.; ut fiat pilulæ, dt. tal. doses, No. vi. S. One pill every morning and evening.

Under this medication the hemorrhage immediately decreases, and soon stops entirely. The recommended mode of treatment is particularly indicated when the hemorrhage is caused by remaining parts of the placenta or fœtus, or when it is owing to an atonic condition of the uterus, the principal object of treatment being then to excite energetic contractions of the uterus. If there are symptoms of chlorosis, Dr. Beau prescribes, after the cessation of the hemorrhage, iron in combination with small doses of ruta.—(*Union Médicale*, No. 7, 1859.)

V.—*A Convenient Mode of Treating Vaginitis and Superficial Inflammation of the Cervix Uteri.* By M. FOUCHER.

M. Foucher mentions, in the *Bulletin de Thérapeutique* of the 15th ult., that in the above affections he prefers ointments to injections. In simple vaginitis, he introduces every morning, with the assistance of the speculum, a good-sized pledget of cotton wool, well smeared over with tannin ointment, into the vagina, bringing the pledget in contact with the cervix. By means of a thread tied to it the wool can be removed by the patient, either in the evening or on the next morning. Every time the pledget is taken off an injection of cold water, or of a solution of alum, should be used to wash the mucous membrane of the vagina. By a little practice patients soon learn to introduce the pledget themselves, the surgeon then cauterizing the inflamed surfaces to hasten cicatrization.

M. Foucher uses the same treatment for fluor albus with much success; but he tries, at the same time, to modify the morbidly disposed organism with the following pills: Extract of rhubarb, quinine or extract of bark, steel reduced by hydrogen, of each half a drachm—for forty pills. To counteract constipation, the author has found half a grain of powdered belladonna, given every night in the form of pill, extremely useful.—(*London Lancet*, August, 1859.)

VI.—*On the Influence of Sex on Diseases of Children.* By Dr. Robert Küttner, of Dresden.

That sex exercises a decided influence upon diseases and their course long before the development of puberty, is well evinced in early infancy. It is one of the important tasks of pathology to study this phenomenon more closely, to establish the facts having relation to it by accurate statistics, and to find out, as far as possible, the real cause of the same. Dr. Küttner's treatise is a valuable contribution toward the solution of this question, as his statements are founded upon the statistics of ten thousand cases treated in the Children's Hospital of Dresden, during a period of over ten years. Referring our readers for particular data to the treatise itself, we only give the conclusions which the author lays down as the result of his statistical researches. They are the following:—

1. Boys are, particularly in the first year of their life, much more liable to diseases of the digestive organs than girls; they bear therefore an improper mode of feeding less easily, and die, the relative mortality of both sexes being equal, in an absolutely greater number of diseases of this kind.
2. Nervous and cerebral diseases are, especially from the fifth year, nearly twice as frequent in boys as in girls.
3. Boys are more disposed to umbilical and inguinal hernia than girls.
4. Girls, after their third, and particularly after their fifth year, are more inclined to diseases of the respiratory organs, and die of them in greater number.
5. The same is the case in regard to diseases of the heart.
6. In acute diseases of the blood the difference of sex does not seem to exercise any influence; chronic anæmia, however, and scorbutic cachexia, are much more frequent in girls than in boys, especially after the eighth year of life, (in the proportion of ten to one.) Scrofula and tuberculosis are, at the beginning, nearly equal; but from the fifth year pulmonary tuberculosis is more frequently met with in girls. Rachitis occurs in equal number in both sexes, but is often somewhat later developed in girls, and is of longer duration in them than in boys.
7. Chronic diseases of the skin (particularly of the scalp) are, after the ninth year, more frequent in girls than in boys.
8. The same is the case in regard to swellings of the thyroid gland.—(*Journal für Kinderkrankheiten*, Nos. 1 and 2, 1859.)

VII.—*Prophylactic Treatment of the Sequelæ of Measles and Scarlet Fever.* By Prof. Scoutetten.

M. Scoutetten, chief physician of the Military Hospital of Metz, advises frictions with warm sweet-oil all over the body, the face included, when the redness is gone. After this friction the patient is replaced in bed, where he should remain about two hours. The next day he should take a warm bath, and remain in it one hour. After the bath he is replaced in bed, and two or three hours afterwards, when the skin is quite dry, another friction is made similar to the first. This is generally sufficient to ward off the unpleasant and well-known sequelæ of the two above-named exanthematous fevers; but when the attack has been violent, and the dead epidermis has not completely fallen off, these means should be resumed until the dermis has regained its functions. M. Scoutetten has rarely gone beyond four frictions and two baths. These precautions being taken, the patients can, according to the author's experience, go out with impunity.—(*Gazette Hebdomadaire*, April, 1859; *Lancet.*)

### VIII.—*On the Treatment of Hooping-cough.* By DR. J. WHITEHEAD.

It is too generally assumed that, except by change of air, hooping-cough cannot be modified by any treatment to a perceptible degree; that it is, at least, possible to abbreviate its course, is proved by the following statistics:—

In thirty-five cases of hooping-cough, admitted to the hospital clinics at Manchester, after an average duration of the disease of more than three months, a cure was obtained in less than twenty-five days at an average; and it is probable that not much more time would have been required, in order to obtain the same result, if the children had entered the hospital six or eight weeks sooner. That such would have been the case is proved by the fact that in eighty-seven cases in which the treatment was commenced after the second week of the disease, the average duration of the treatment was the same as in the first series of patients; the duration of the disease was thus reduced to thirty-seven days. The average duration of the disease was forty-two days in the totality of the cases, and one hundred and eleven days in those cases which had been neglected.

Among the eighty-seven cases of the second series, there were thirty-two in whom the average duration of the disease, at the time of their admission, had been eleven days; and fifty-five in whom it had been five days. In the former, the average duration of the whole disease was thirty-five days; in the latter, but thirty-two days.

The treatment employed consisted, in simple cases and in such in which the existing complications had been removed, in the administration of Dover's powder, either alone or combined with camphor, which was used either internally or in fumigations; in the use of emetics, belladonna, and of revulsives. In all cases opium or belladonna served as the basis of the treatment.—(*Third Report of the Clinical Hospital*, Manchester, 1859, p. 87, *et seq.*)

### IX.—*On the Curability of Tubercular Meningitis.* By DR. O. BANG.

Although many physicians deny the curability of tubercular meningitis, (hydrocephalus acutus,) as, for instance, Camper and Trousseau, some others have proved from their own experience that a cure may be sometimes obtained, though such cases be of very rare occurrence. West saved, of thirty-four children, one; Guersant, of an hundred in the second stage, one; in the third stage, however, none. Rilliet, who has collected and compared all the cases of cure in the *Arch. Générales*, (December, 1853,) doubts many of the favorable cases, and admits only eight cases reported by others, and three observed by himself to have been real cases of tubercular meningitis. To these cases Liégard (*sur la nature et le traitement de la fièvre cérébrale*) adds a case of his own, and six observed by his father; the total number of cured cases on record are thus eighteen.

Those who maintain the incurability of the affection suppose that in the cases reported as cured, simple inflammation of the brain, or remittent fever, worm-fever, hydrocephaloid, etc. had been mistaken for tubercular meningitis. The author believes that an exact differential diagnosis between simple and tubercular meningitis can hardly be established. According to his opinion, meningitis consists in an active congestion, followed by an inflammation which assumes a different character when the blood is healthy, and when it is diseased; in the former case the disease is simple, in the latter it is complicated. *The tubercular meningitis is characterized by its occurrence in scrofulous children;* the meningitis is here complicated with a disease of the blood, and is thus to be treated according to the same rules which are observed in the treatment of any inflammation accompanied by a morbid condition of the blood. A complete cure is possible only when an amelioration of the crasis of the blood can be effected, and this is very difficult after a localization of the disease has taken place. Only if a meningitis appears suddenly without premonitory symptoms, and is accompanied by violent fever, abstraction of a considerable quantity of blood is necessary; the use of this measure requires, however, great caution when the disease commences more slowly, and the child bears the characters of the scrofulous cachexia.

A prophylactic treatment is indicated only in cases of the latter kind; it consists in the use of anti-scrofulous remedies and of derivatives, when the children have a disposition to congestion of the brain; at the same time all strong impressions upon the brain should be carefully avoided.

The author has treated three children in this manner, who had lost their brothers of tubercular meningitis, and in whom certain symptoms threatened an attack of the disease; blisters to the nape of the neck were of particular use in this instance. If the parents are scrofulous, or if there is a hereditary tendency in the family, Dr. Bang subjects the women to an anti-scrofulous treatment during their pregnancy, and has found this plan very successful in one case. Cold affusions upon the head while the child is sitting in a warm bath are very useful, not only as prophylactic, but also as curative means; if, however, the state of exudation has commenced, he uses, instead of them, powerful derivatives upon the shaven head, such as blisters, croton oil, ointment of tartar emetic, etc. In three cases in which considerable exudation had already taken place, the cure was very much promoted by an ointment composed of unguentum hydrargyri, tartar emetic, and ol. tiglii. In simple as well as in tubercular hydrocephalus, calomel, in doses of three to eight grains, is the most useful laxative; in chronic cases the author gives it, however, in smaller doses in combination with digitalis, as an antiphlogistic. As calomel seemed to promote vomiting, the author prescribed frictions with mercurial ointment on different parts of the body; this application was employed in two cases, which were cured. Two cases were treated with iodine in combination with other remedies, and also in these a cure was obtained by the author; in other cases, in which he used iodine alone, it seemed to diminish vomiting, but did not effect a cure.

In accordance with this experience the author considers tubercular meningitis curable, and is supported in his opinion by cases reported by reliable observers, in which all the symptoms of the disease were present, and which were, nevertheless, cured. If the physician is called in early enough, and follows the plan of treatment recommended above, there is, according to the author, hope of the disease being cured.—(*Bibliothek für Laeger*, No. 7, p. 241; *Schmidt's Jahrbücher*, May, 1859.)

X.—*Defective Assimilation in Infants; its Prevention and Treatment.* By DR. ROUTH. (Read before the Medical Society of London.)

The object of this paper was to show that most of the mortality of infants was due to defective assimilation. Defective assimilation was almost always the result of want of breast milk, and the use of injudicious food; the disease was most effectively prevented by supplying this milk. Dr. Routh then detailed the result of breast milk exclusively given, artificial food without breast milk and with it, on the development and mortality of children, from tables of Drs. Merei and Whitehead; from which he showed that in proportion as breast milk predominated, in proportion was good development observed, and *vice versa*. He then showed that the most frequent diseases among children were abdominal diseases, occurring in the proportion of 23·4 per cent.; developmental diseases in that of 8·8 per cent. of all cases; rachitic diseases constituting 3·2 per cent.; atrophy or marasmus, 5·2 per cent. He believed, however, that all these were produced by defective assimilation, the former, in most cases, being sequelæ of it, atrophy or marasmus being only the more marked and characteristic stage.

Dr. Routh then described the disease as consisting of three stages: First, or premonitory, in which peevishness, some loss of flesh, occasional attacks of indigestion, acid eructations, etc. were most prevalent; in the second stage, *emaciation* was more marked, eyes became unusually bright, much loss of digestive power, sometimes with diarrhœa and lientery; third, or exhaustive stage, generally attended with diarrhœa, aphthæ, frightful emaciation, complete loss of digestion, etc. Sometimes the disease, from the second stage, passed on to tuberculosis, rachitism, and most developmental disorders, and not to the third stage.

*Causes.*—The predisposing causes were hereditary, tubercular habit, and exanthemata. Exciting causes—bad air, want of cleanliness, injudicious food, and especially an atmosphere contaminated by too many children being congregated together.

*Post-mortem appearances.*—Three kinds: Emaciation very great, loss of adipose, cellular, and muscular tissue, in all varieties; but in one, where diarrhœa had been present, red patches, or aphthæ over the alimentary mucous membrane, these aphthæ containing the oïdium albicans.

In other cases, also with diarrhœa, the mucous membrane exuding a reddish-colored mucus, intensely acid. In others, without diarrhœa or with it, Peyer's glands projecting, and enlarged in patches, as in Asiatic cholera. In all, undigested matter in the canal, with very fetid fæcal matters.

The disease seems to be gradual, passing on to entire loss of *primary* assimilation; the secondary still persisting, although inactive from want of assimilable matters to take up. Albuminous, starchy, and oily matters were not digested.

The *treatment* consists in supplying fatty acids, and artificially-digested animal and occasionally vegetable substances, especially human milk. If this could not be sucked, it should be collected in a cup and given by the spoon. Dr. Routh strongly animadverted here upon the absurd dogma, that it is wrong to mix human and cow's milk. He, on the contrary, believed the plan not only safe, but the very best practice in many cases, and the only means of saving an infant's life. Simple juice of meat, and this with vegeto-animal food, he had found most useful in fulfilling these indications. The remedies were of two kinds: 1st. Those calculated to increase cell growth and development. Phosphate of soda, producing an emulsion with fats, thus allowing of their assimilation; chloride of potassium, to dissolve carbonate of lime; phosphate of lime, to enable blood to take up more carbonic acid, and thus hold in solution more carbonate of lime; (these substances severally strengthening muscular and bony structure;) lime-water, to provide lime to blood. 2d. These last also acted as some of the remedies calculated to allay local irritation of the alimentary canal. Carminatives were useful, such as dill, but especially cinnamon-powder, to correct flatus and to check diarrhœa. Anodynes were also (however objected to generally) strongly recommended by the author. For the diarrhœa, when present, nitrate of silver and sulphate of copper were the best remedies. Wine was also found very serviceable, even if given in large quantities. These remedies, however, it must be confessed, proved in most cases of no avail in the third stage, which was, he might say, almost incurable; but they acted very effectively in the second and first stages.—(*Lancet*, June 18, 1859.)

